# Structural Characterization and Identification of Major Constituents in Jitai Tablets by High-Performance Liquid Chromatography/Diode-Array Detection Coupled with Electrospray Ionization Tandem Mass Spectrometry

**DOI:** 10.3390/molecules170910470

**Published:** 2012-09-03

**Authors:** Shuping Wang, Lei Liu, Lingling Wang, Yaohua Hu, Weidong Zhang, Runhui Liu

**Affiliations:** 1 School of Pharmacy, Second Military Medical University, Shanghai 200433, China; Email: shupingwang2007@163.com (S.W.); huyaohua2004@126.com (Y.H.); 2 College of Pharmacy, Nankai University, Tianjin 300071, China; Email: mark_2004@163.com; 3 National Engineering Research Center for TCM, Shanghai 201203, China; Email: pharmacy2001@163.com; 4 School of Pharmacy, Shanghai Jiao Tong University, Shanghai 200240, China

**Keywords:** constituents identification, TCM, HPLC-DAD/ESI-MS/MS, Jitai tablet

## Abstract

In the present study a universally applicable HPLC-DAD/ESI-MS/MS method was developed for carrying out the comprehensive characterization of Jitai tablets (JTT). Based on the ESI-MS^n^ fragmentation patterns of the reference standards, a total of 101 components were identified or tentatively characterized by comparing their retention times, UV and MS spectra with those of reference standards or through the matching of empirical information with those of published components in the in-house library. The characteristic fragmentation pattern of alkaloids, phenolic acids, tanshinones, flavonoid glycosides, cyanogenic glycosides, ginsenosides, 2-(2-phenylethyl) chromones, phthalides and gingerol-related compounds were tentatively elucidated using structurally-relevant product ions. It was observed that neutral losses of C_9_H_10_O_3_ and C_9_H_8_O_2_ were the characteristic product ions of scopola alkaloids. Neutral fragment mandelonitrile was the characteristic ion of cyanogenic glycosides. To our knowledge, tropylium ion and C_4_H_2_O unit were the characteristic ions of 2-(2-phenylethyl) chromone, which resulted from the Retro-Diels-Alder (RDA) cleavage of the C ring. The results indicated that the developed analysis method could be employed as a rapid, effective technique for structural characterization of chemical constituents in TCM. This work is expected to provide comprehensive information for the quality evaluation and pharmacokinetic studies of JTT.

## 1. Introduction

In recent years, Traditional Chinese Medicines (TCMs) have been attracting considerable attention worldwide for their complementary therapeutic effects to Western drugs, but with low toxicity, in treating drug addiction, especially opiate addiction. Jitai tablets (JTT), which have been already approved for the treatment of opiate addiction by the Chinese State Food and Drug Administration (SFDA), are prepared from 15 medicinal materials, including *Rhizoma Corydalis*, *Radix Salviae Miltiorrhiae*, *Radix Angelicae sinensis*, *Rhizoma Chuanxiong*, *Semen Persicae*, *Flos Carthami*, *Radix Aconite*, *Radix Ginseng*, *Cortex Cinnamomi*, *Rhizoma Zingiberis*, *Semen Myristicae*, *Flos Daturae*, *Radix Aucklandiae*, *Lignum Aquilariae Resinatrm* and *Margarita*. JTT have been proved to be very safe and effective in the inhibition of protracted withdrawal symptoms with less harmful side effects [1,2], and good for the rehabilitation of abnormal body functions induced by chronic drug use, including improving immune function, increasing working memory and preventing neurological disorders [3,4]. Despite so many beneficial effects, there is no integrated study on the chemical constituents of JTT, not only due to the complexity of the formula, but also the unreality and impracticality of basing such studies on reference standards. It is generally considered that the beneficial effects of TCMs are mainly due to the synergistic effects of constituents in the medicines. Hence, it is imperative to establish sensitive and comprehensive analytical methods to acquire a better understanding of the material basis and to enhance the product quality control.

Up to now, high performance liquid chromatography-diode array detection (HPLC-DAD) coupled with electrospray ionization tandem mass spectrometry (ESI-MS/MS) has become one of the most popular methods applied for identification and elucidation of components in complex TCM preparations [5,6,7,8,9,10]. Benefitting from using the fragmentation patterns and the comparison of the UV, MS data with those of reference standards and literature data, even the unknown constituents could be interpreted [11,12,13]. Herein is described an extensive investigation of fragmentation pathways leading to the identification of key diagnostic fragment ions for the multiple constituents originated from JTT. On the basis of extracted ion chromatograms (EIC), the elemental compositions and the complementary multilevel structural information of 101 components originated from JTT were tentatively elucidated and confirmed by comparing their retention behaviors and MS spectra with supporting results on the fragmentation path way of 23 reference standards and literature data. To our knowledge, this is the first integrative study on characterizing the multiple constituents in JTT, the developed analysis method could be employed as a rapid, effective technique for structural characterization of chemical constituents in TCM. This work is expected to provide comprehensive information for the quality evaluation and pharmacokinetic studies of JTT.

## 2. Results and Discussion

**Figure 1:**
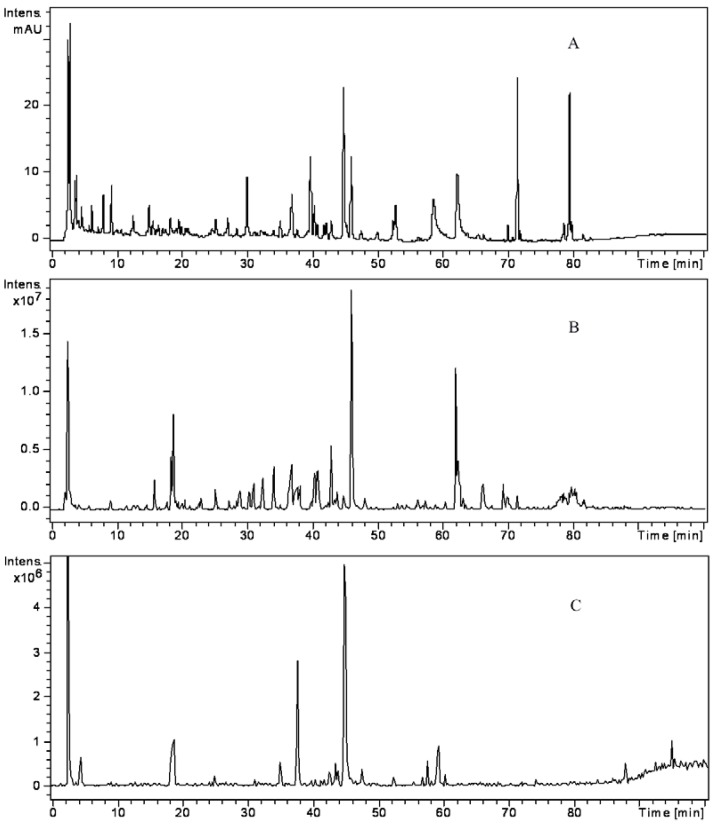
UV chromatogram obtained at 280 nm (**A**), LC-ESI-MS base peak chromatogram (BPC) of JTT in positive (**B**) and negative (**C**) ion mode.

All the constituents were identified by the interpretation of their mass spectral behavior obtained from HPLC-DAD/ESI-MS/MS spectra and also taking into account the data provided by the 23 reference standards and literature. Figure 1 shows the chromatogram of JTT registered at 280 nm (**A**), the HPLC-ESI-MS/MS base peak chromatogram (BPC) of JTT in positive (**B**) and negative (**C**) ion mode. 

**Figure 2:**
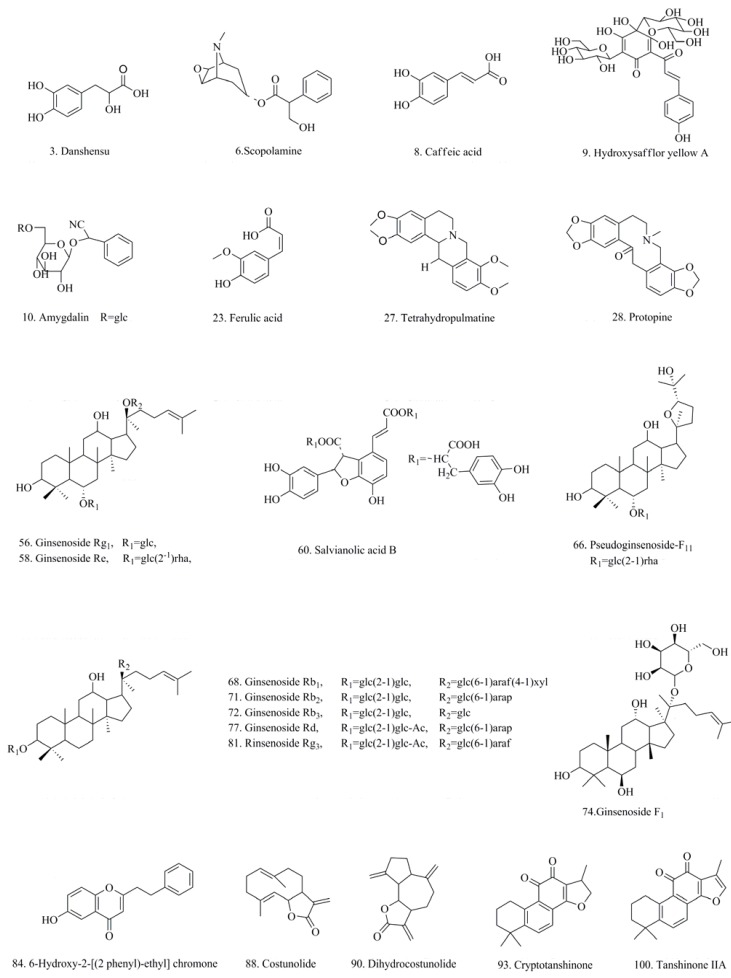
Chemical structures of the reference standards.

Figure 2 shows the structures of the reference standards. Table 1 shows the identification results of JTT by HPLC-DAD/ESI-MS/MS. MS and MS^n^ (n = 2–4) data of these reference standards were obtained by collision-induced dissociation (CID). 

**Table 1:** The identification results of JTT by HPLC-ESI-MS/MS.

Peak No.	*t*_R _(min)	λ_max_ (nm)	M.W	MS and MS^n^ fragments (%)		Name	Origin
				MS ^+^ ( *m/z*)	MS ^−^ ( *m/z*)		
1	11.7	230, 406	485.3	MS^2^[486]: 436(100), 404(44)		Mesaconine	1
				MS^3^[486→436]: 404(100), 372(22), 293(21)			
2	13.4	228, 260, 294	154.1		MS^2^[153]: 109(100)	Procatechuic acid	2
3	14.8	280, 288	198.1		MS^2^[395]: 197(100), 179(2)	Danshensu ^a^	2
4	15.5	276, 339	788.7		MS^2^[787]: 625(100), 301(2), 463(3)	6-Hydroxy kaempferol (3 × Glc/Gal)	3
5	16.0	266, 347	802.2		MS^2^[801]: 625(100), 668(3), 463(3)	6-Hydroxy kaempferol (2 × Glc + GluA)	3
					MS^3^[801→625]: 463(100), 301(31)		
6	16.8		303.1	MS^2^[304]: 156(78), 138(100), 110(22)		Scopolamine ^a^	12
7	17.7	256, 352	640.5		MS^2^[639]: 463(100), 301(6)	Quercetin (Glc/Gal + GluA)	3
					MS^3^[639→463]: 301(100), 271(10)		
8	18.0	295, 324	180.1	MS^2^[180]: 163(100)	MS^2^[179]: 135(100)	Caffeic acid ^a^	2
9	18.4	225, 403	612.2		MS^2^[611]: 491(100), 403(28), 473(7)	Hydroxysafflor yellow A ^a^	3
10	18.8		457.1		MS^2^[502]: 456(100), 323(8),	Amygdalin ^a^	11
					MS^3^[502→456]: 323(100), 221(11), 179(8)		
11	19.1	256, 350	626.5		MS^2^[625]: 463(100), 557(11), 577(10), 301(9)	Quercetin 2 × Glc/Gal	3
					MS^3^[625→463]: 301(100), 254(2)		
12	19.8	280, 340	934.8		MS^2^[933]: 771(100), 625(21) MS^3^[933→771]: 609(100), 301(7), 463(5)	6-Hydroxy kaempferol (2 × Glc/Gal + Rut)	3
13	19.5	266, 340	640.1		MS^2^[639]: 463(100), 571(38), MS^3^[639→463]: 301(100), 256(3)	6-Hydroxy kaempferol (Glc/Gal + GluA)	3
14	21.0	276, 339	624.1		MS^2^[623]: 447(100), MS^3^[623→477]: 285(100)	Kaempferol (Glc/Gal + GluA)	3
15	22.6		289.1	MS^2^[290]: 221(100), 124(30) MS^3^[290→221]: 203(100)		Atropine	12
16	23.5		295.2		MS^2^[340]: 294(100), 161(22)	Prunasin	11
17	24.0	271, 335	758.2		MS^2^[757]: 595(100), 287(9) MS^3^[757→595]: 287(100), 329(5)	Carthamidin (Glc/Gal + Rut)	3
18	24.4	252, 332	334.3	MS^2^[335]: 317(100), 299(18) MS^3^[335→317]: 299(100)	MS^2^[333]: 315(100), 297(15) MS^3^[333→315]: 297(100)	5,6,7,8-Tetrahydroxy-tetrahydro-2-[2-(4'-hydroxyphenyl)-ethyl]chromone	6
19	24.7	265, 347	772.2		MS^2^[771]: 609(100), 463(15) MS^3^[771→609]: 301(100)	6-Hydroxykaempferol (Glc/Gal + Rut)	3
20	25.0	265, 347	626.5		MS^2^[625]: 463(100), 301(33) MS^3^[625→463]: 301(100)	6-Hydroxykaempferol (2 × Glc/Gal)	3
21	28.1	271, 335	758.2		MS^2^[757]: 449(100), 287(28) MS^3^[757→449]: 287(100)	Carthamidin (Glc/Gal + Rut)	3
22	28.6	253, 336	318.1	MS^2^[319]: 301(100), 283(22), 255(7) MS^3^[319→301]: 287(100)	MS^2^[363]: 317(100), 299(25), 281(6) MS^3^[363→317]: 281(100)	Isoagarotetrol	6
23	30.1	238, 280, 322	194.1	MS^2^[177]: 145(100)	MS^2^[193]: 178(100)	Ferulic acid ^a^	2
24	30.4	280	341.1	MS^2^[342]: 178(100), 163(21) MS^3^[342→178]: 163		Tetrahydrocolumbamine	10
25	31.7	253, 339	318.1	MS^2^[319]: 301(100), 283(25), 255(7) MS^3^[319→301]: 283(100)	MS^2^[363]: 317(100), 299(23), 281(6) MS^3^[363→317]: 281(100)	Agarotetrol	6
26	32.4	256, 353	593.2		MS^2^[592]: 472(100), 364(21), 446(14) MS^3^[592→472]: 244(100), 364(42)	Hydroxyl cartormin	3
27	32.5	280	355.1	MS^2^[356]: 192(100), 165(13)		Tetrahydropulmatine ^a^	10
				MS^3^[356→192]: 177(100)			
28	34.1	285	353.1	MS^2^[354]: 188(100), 149(93), 159(8)		Protopine ^a^	10
				MS^3^[354→188]: 159(100)			
29	34.3	270, 335	450.1		MS^2^[449]: 287(100), 259(5)	Carthamidin/isocarthamidin (Glc/Gal)	3
					MS^3^[449→287]: 259(100)		
30	34.9	256, 353	592.2		MS^2^[593]: 285(100), 257(22)	Kaempferol-3-O-Rutinoside	3
					MS^3^[593→285]: 257(100)		
31	35.4	230, 403	1044.3		MS^2^[1043]: 1026(100), 863(7)	Anhydrosafflor yellow B	3
					MS^3^[1043→1026]: 863(100)		
32	36.5	254, 288	538.1		MS^2^[537]: 339(100), 295(17)	Salvianolic acid I/H	2
					MS^3^[537→339]: 295(100)		
33	36.8	280	355.7	MS^2^[356]: 325(100), 294(7)		Glaucine	10
				MS^3^[356→325]: 294(100)			
34	36.9	280	338.3	MS^2^[338]: 323(100), 294(8)		Columbamine	10
				MS^3^[338→323]: 294(100)			
35	37.1	238, 330	340.1		MS^2^[339]: 295(100), 277(8)	Salvianolic acid G	2
					MS^3^[339→295]: 277(100)		
36	37.2	248, 328	418.3		MS^2^[417]: 209(100), 194(22)	Salvianolic acid D	2
					MS^3^[417→209]: 194(100)		
37	37.6	234, 288	716.3		MS^2^[715]: 393(100), 257(5)	Dedihydro-salvianolic acid B/isomer	2
					MS^3^[715→393]: 257(100)		
38	37.7	277	320.3	MS^2^[320]: 292(100), 302(50)		Coptisine	10
				MS^3^[320→292]: 292(100), 275(75)			
39	38.1	240	589.3	MS^2^[590]: 540(100), 508(5)		Benzoylmesaconitine	1
				MS^3^[590→540]: 508(100)			
40	38.5	270, 340	575.2		MS^2^[574]: 454(100), 304(15)	Cartormin	3
					MS^3^[574→454]: 304(100)		
41	38.6	288	323.1	MS^2^[324]: 149(100), 176(5)		Tetrahydrocoptisine	10
				MS^3^[324→149]: 176(100)			
42	39.7	275	224.2	MS^2^[207]: 165(100), 135(7)		Senkyunolide I	6/7
				MS^3^[207→165]: 135(100)			
43	39.7	238, 330	718.1		MS^2^[717]: 519(100), 321(6)	Salvianolic acid E	2
					MS^3^[717→519]: 321(100)		
44	40.0	285	340.1	MS^2^[340]: 176(100), 149(16)		Tetrahydroberineper	10
				MS^3^[340→176]: 119(100)			
45	40.3	224, 328	360.1		MS^2^[359]: 161(100), 133(12)	Rosmarimic acid	2
					MS^3^[359→161]: 133(100)		
46	40.4	280	369.4	MS^2^[370]: 165(89),192(100)		Corydaline	10
				MS^3^[370→192]: 149(100), 177(85)			
47	40.5	232, 254, 310	538.1	MS^2^[556]: 341(100), 295(5)	MS^2^[537]: 493(100), 295(19)	Lithospermic acid	2
				MS^3^[556→341]: 295(100)	MS^3^[537→493]: 295(100)		
48	41.0	243	603.3	MS^2^[604]: 586(100), 554(33), 522(7)		Benzoylaconine	1
				MS^3^[604→586]: 554(100), 522(33)			
49	41.3	275	224.2	MS^2^[207]: 189(100), 145(15)		Senkyunolide H	6/7
				MS^3^[207→189]: 145(100)			
50	41.7		933.1		MS^2^[932]: 799(8), 637(100), 475(3)	Notoginsenoside R1	4
					MS^3^[932→637]: 475(100)		
51	41.7	232, 288	494.1		MS^2^[493]: 295(100), 159(17)	Salvianolic acid A	2
					MS^3^[493→295]: 159(100)		
52	42.6	220, 235, 280	750.2		MS^2^[749]: 339(100), 321(8)	8-Hydroxy-9''-methyl-, salvianolate B	2
					MS^3^[749→339]: 321(100)		
53	42.9	275	352.1	MS^2^[352]: 337(100), 308(32)		Palmatine	10
				MS^3^[352→337]: 308(100)			
54	43.2	228, 403	614.2		MS^2^[613]: 551(100), 533(15)	Safflomin C	3
					MS^3^[613→551]: 533		
55	43.4	235, 405	573.3	MS^2^[574]: 542(100), 510(15)		Benzoylhypoaconine	1
				MS^3^[574→542]: 510(100)			
56	43.6		801.1		MS^2^[846]: 800(100), 637(23), 475(18)	Ginsenoside Rg1a	4
					MS^3^[846→800]: 637(100), 475(5)		
57	43.8	277	336.3	MS^2^[366]: 321(100), 292(5)		Berberine	10
				MS^3^[366→321]: 292			
58	43.9		947.1		MS^2^[946]: 637(100), 475(5)	Ginsenoside Re ^a^	4
					MS^3^[946→637]: 475(100)		
59	44.6	220, 235, 280	750.2		MS^2^[749]: 551(100), 321(18)	8-Hydroxy-9'''-methyl-, salvianolate B	2
					MS^3^[749→551]: 321(100)		
60	44.7	216, 234, 288	718.1		MS^2^[717]: 519(100), 321(6)	Salvianolic acid B ^a^	2
					MS^3^[717→519]: 321(100)		
61	46.0	265	366.1	MS^2^[366]: 351(100), 322(22)		Dehydrocorydaline	10
				MS^3^[366→351]: 322(100)			
62	49.4	225, 240, 280	732.2		MS^2^[731]: 533(100), 335(21)	4-Methoxyl-salvianolic acid B	2
					MS^3^[731→533]: 335(100)		
63	50.1		1241.4		MS^2^[1240]: 1107(100), 945(22), 783(7)	Ginsenoside Ra3/notoginsenoside F	4
					MS^3^[1240→1107]: 945(100), 783(25)		
64	52.2	224, 288, 324	492.1		MS^2^[491]: 293(100), 265(33), 249(20)	Salvianolic acid C	2
					MS^3^[491→293]: 265(100), 249(12)		
65	52.3	230, 280, 425	294.5	MS^2^[295]: 177(100), 145(5)	MS^2^[293]: 193(100), 179(42)	6-Gingerol	5
				MS^3^[295→177]: 145(100)	MS^3^[293→193]: 179(100)		
66	56.7		801.1		MS^2^[800]: 637(100), 475(25)	Pseudoginsenoside-F11 ^a^	4
					MS^3^[800→637]: 475(100)		
67	57.2		1211.3		MS^2^[1210]: 1078(100), 945(13)	Ginsenoside Fc/Ra1/Ra2	4
					MS^3^[1210→1078]: 945(100)		
68	57.4		1109.3		MS^2^[1108]: 946(100), 784(7)	Ginsenoside Rb1 ^a^	4
					MS^3^[1108→946]: 784(100)		
69	57.8		770.9		MS^2^[815]: 770(100), 637(12), 475(5)	Notoginsenoside R2	4
					MS^3^[815→770]: 637(100), 475(26)		
70	58.0		1151.3		MS^2^[1150]: 1108(100), 946(6)	Quinquenoside R1	4
					MS^3^[1150→1108]: 946(100)		
71	58.2		1079.3		MS^2^[1078]: 945(100), 783(22), 621(31), 459(7)	Ginsenoside Rb2 ^a^	4
					MS^3^[1078→945]: 783(100)		
72	58.5		1079.2		MS^2^[1078]: 945(100), 783(56), 621(19), 459(4)	Ginsenoside Rb3 ^a^	4
					MS^3^[1078→945]: 783(100), 621(21)		
73	58.7		1121.3		MS^2^[1120]: 1078(100), 945(12)	Ginsenoside Rs1/Rs2	4
					MS^3^[1120→1078]: 945(100)		
74	59.0		638.4		MS^2^[683]: 637(100)	Ginsenoside F1 ^a^	4
75	59.2		1121.3		MS^2^[1120]: 1078(100), 945(44)	Ginsenoside Rs1/Rs2	4
					MS^3^[1120→1078]: 945(100)		
76	59.4		957.1		MS^2^[956]: 794(100), 613(6), MS^3^[956→794]: 613(100)	Ginsenoside Ro	4
77	60.0		947.1		MS^2^[946]: 784(100), 621(45), 459(7)	Ginsenoside Rd ^a^	4
78	60.7		1033.3		MS^2^[1032]:, 988(16), 946(100), 784(22), 621(4)	Malonyl ginsenoside Rd	4
					MS^3^[1032→946]: 784(100)		
79	63.2	280	192.1	MS^2^[193]: 147(100), 175(22)		Senkyunolide A	6/7
				MS^3^[193→147]: 105(100)			
80	63.7	242, 349	296.3	MS^2^[297]: 206(100), 140(24)	MS^2^[295]: 204(100), 138(22)	6-Hydroxy-7-methoxy-2-[(2-phenyl)-ethyl]chromone	6
				MS^3^[297→206]: 140(100)	MS^3^[295→204]: 138(100)		
81	64.0		785.2		MS^2^[784]: 621(100), 475(32)	Ginsenoside Rg3 ^a^	4
					MS^3^[784→621]: 475(100)		
82	64.5	225, 277, 425	308.6	MS^2^[291]: 191(100), 176(34)		Methyl-[6]-gingerol	5
				MS^3^[291→191]: 176(100)			
83	65.1	240, 260, 310	188.1	MS^2^[189]: 171(100), 143(5)		E-butenyl phthalide	6/7
				MS^3^[189→171]: 143(100)			
84	65.6	227, 330	266.1	MS^2^[267]: 176(100), 110(12)	MS^2^[265]: 174(100), 146(17)	6-Hydroxy-2-[(2 phenyl)-ethyl] chromone^ a^	6
				MS^3^[267→176]: 110(100)	MS^3^[265→174]: 146(100)		
85	67.8	230, 280, 425	322.3	MS^2^[305]: 177(100), 145(7)	MS^2^[321]: 193(100), 178(6)	8-Gingerol	5
				MS^3^[305→177]: 145(100)			
86	68.9	328, 240	310.1	MS^2^[311]: 220(100), 205(45)	MS^2^[309]: 218(100), 203(13)	6, 7-Dimethoxy-2-[(2-phenyl)-ethyl]chromone	6
				MS^3^[311→220]: 205(100)	MS^3^[309→218]: 203(100)		
87	71.2	206, 280, 326	190.2	MS^2^[191]: 173(100), 145(22)		Z-ligustilide	6/7
				MS^3^[191→173]: 145(100)			
88	72.1	225	232.1	MS^2^[233]: 215(100), 187(14)		Costunolide ^a^	8
				MS^3^[233→215]: 187(100)			
89	72.9	225, 275, 420	276.2	MS^2^[277]: 137(100), 122(5)		6-Shogaol	5
				MS^3^[277→137]: 122(100)			
90	73.5	230	230.1	MS^2^[231]: 213(100), 185(42)		Dihydrocostunolide ^a^	8
				MS^3^[231→213]: 185(100)			
91	74.1	230, 275, 422	338.3	MS^2^[321]: 163(100), 131(24)		3- or 5-Acetoxy-[6]-gingerdiol	5
				MS^3^[321→163]: 131(100)			
92	74.5	230, 275, 422	380.2	MS^2^[398]: 261(100), 163(13)		Diacetoxy-[6]-gingerdiol	5
				MS^3^[398→261]: 163(100)			
93	75.1	218, 264, 360	296.1	MS^2^[297]: 253(100), 211(11)		Cryptotanshinone ^a^	2
				MS^3^[297→253]: 211(100)			
94	76.4	250, 270,354	294.1	MS^2^[295]: 277(100), 249(19)		Isotanshinone IIA	2
				MS^3^[295→277]: 249(100)			
95	77.2	230, 282, 430	350.4	MS^2^[333]: 177(100)	MS^2^[349]: 193(100), 178(15)	10-Gingerol	5
					MS^3^[349→193]: 178(100)		
96	78.4	280	380.1	MS^2^[381]: 191(100), 173(24)		Tokinolide B	6/7
				MS^3^[381→191]:173(100), 155(21)			
97	79.0	232, 280, 425	394.2	MS^2^[412]: 275(100), 177(44)		Methyl diacetoxy -[6]-gingerdiol	5
				MS^3^[412→275]: 177(100)			
98	81.1	280	380.1	MS^2^[381]: 191(100), 173(11),		Riligustilide	6/7
				MS^3^[381→191]: 173(100), 155(42)			
99	81.6	280	380.1	MS^2^[381]: 191(100), 173(51),		Levistolide A	6/7
				MS^3^[381→191]: 173(100), 155(12)			
100	82.6	270, 354	294.1	MS^2^[295]: 277(100), 249(14)		Tanshinone IIA ^a^	2
				MS^3^[295→277]:249(100)			
101	87.0	225, 275, 420	332.3	MS^2^[333]: 137(100), 122(23)		10-Shogaol	5
				MS^3^[333→137]: 122(100)			

Origin: 1: *Radix Aconiti Lateralis Preparata*; 2: *Radix Salviae Miltiorrhiae*; 3: *Flos Carthami*; 4: *Radix Ginseng*; 5: *Rhizoma Zingiberis*; 6: *Rhizoma Chuanxiong*; 7: *Radix Angelicae Sinensis*; 8: *Radix Aucklandiae*; 9: *Lignum Aquilariae Resinatrm*; 10: *Rhizoma Corydalis*; 11: *Semen Persicae*; 12: *Flos Daturae*. ^a^ Structure confirmed by comparison with reference standards.

In the full scan mass spectra, [M−H]^−^ was observed in negative ion ESI mode, [M+H]^+^, [M+H−NH_4_]^+ ^and [M+Na]^+^ were observed in positive ion ESI mode. The information of [M−H]^−^, [M+H]^+^ and [M+Na]^+^ were used to determine molecular weight. The identification of these compounds was carried out referring to their MS and MS^n^ spectra, and the characteristic fragmentation pattern of alkaloids, phenolic acids, tanshinones, flavonoid glycosides, cyanogenic glycosides, ginsenosides, 2-(2-phenylethyl) chromones, phthalides and gingerol-related compounds are summarized as follows. 

### 2.1. Identification of Alkaloids in JTT

Maximum absorptions in the range from 260 to 300 nm are exhibited by alkaloids in their UV spectra, and the preliminary identification could be facilitated by this characteristic absorption [14]. Consequently, 18 peaks with suitable maximum absorptions were detected as alkaloids in JTT. Furthermore, three major types including isoquinoline alkoloids, scopola alkaloids and aconitine-type alkaloids were summarized mainly based on their mass fragmentation behavior.

**Figure 3:**
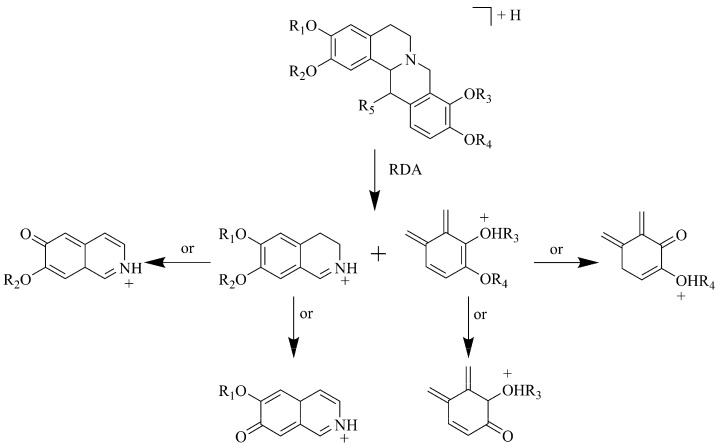
Proposed fragmentation pathway for tetra-proberberine alkaloids in positive mode.

Using the proposed method, 10 isoquinoline alkoloids including four tertiary alkaloids, and six quaternary alkaloids were identified. In the positive ionization mode, the protonated molecule [M+H]^+^ ions of tertiary alkaloids in ESI-MS/MS spectra were observed to undergo the Retro-Diels-Alder (RDA) cleavage. The fragmentation pathway is shown in Figure 3, and the same fragmentation behavior could also be applied for identifying tetra-proberberine alkaloids.

In the positive ionization mode, high abundant product ion [M−CH_3_]^+^ with characteristic *m/z* value were exhibited by quaternary alkaloids. In addition, fragment ions [M−CO−CH_3_]^+^ and [M−2CH_3_]^+^ were observed in the MS/MS spectra, which indicated unique fragments produced by the losses of CO and CH_3_. By using structurally-relevant product ions in literature data [15], six quaternary alkaloids were tentatively identified.

It is reported that the specific fragmentation pathways of the aconitine-type alkaloids were the neutral losses of H_2_O, CO and CH_3_OH in MS^n^ spectra [16]. In the present study, highly abundant protonated molecule [M+H]^+^ ions of peak 1, 39, 48 and 55 were observed in the MS spectra and fragment ions [M+H−H_2_O−CH_3_OH]^+^ and [M+H−H_2_O−2CH_3_OH]^+^ were exhibited in their MS/MSspectra, which indicated a further fragmentation by the neutral losses of CH_3_OH eliminated from the methoxyl on C_16_ position and the proton on the C_15_ position. By using structurally-relevant product ions in literature data [17], they were tentatively identified as mesaconitine, benzoylmesaconitine, benzoylaconine and benzoylhypoaconine, respectively.

For scopola alkaloids, protonated molecular ions [M+H]^+^ were observed in the MS spectra. In the MS/MS spectra, *m/z* 138 [M+H−C_9_H_10_O_3_]^+^ was formed by the loss of tropic acid, while *m/z* 156 [M+H−C_9_H_8_O_2_]^+^ was produced by the loss of C_9_H_8_O_2_ unit resulted from the fragmentation reaction of the ester bond. It could be concluded that the ions at *m/z* 138 and 156 were the characteristic product ions of scopola alkaloids. By comparison with reference standards and literature data [18], the [M+H]^+^ ions of scopolamine and atropine eluting at 16.8 and 22.6 min were tentatively identified, respectively.

### 2.2. Identification of Phenolic Acids in JTT

Phenolic acids exhibited a unique fragmentation pattern in the negative ionization mode. They could be classified into monomers and polymers including dimers, trimers and tetramers, which were calculated by the number of phenyls in the structure accordingly [19]. Highly abundant quasi-molecular [M−H]^−^ ions were first yielded in their MS spectra. In the MS/MS spectra, small molecules such as CO_2_, CO and H_2_O were produced by the monomers, which contained either carboxyl, carbonyl or hydroxyl groups. Moreover, [M−H−C_9_H_10_O_5_]^−^ ion and [M−H−C_9_H_8_O_4_]^−^ ion were obtained via dissociation of the bonds on either side of the esterifiable carboxyl oxygen in polymers, which also showed the same general fragmentation pattern for their quasi-molecular ions [20,21]. This strategy was successfully applied for identifying or tentatively characterizing 16 phenolic acids with the structurally-relevant product ions.

### 2.3. Identification of Tanshinones in JTT

In the positive ionization mode, the protonated molecule [M+H]^+^ ions of tanshinones were observed in the MS spectra. It is reported that the hydrogen at C-1 and oxygen at C-11 of tanshinones were the source of the dissociated H_2_O, the loss of CO, O, CH_3 _and H_2_O were also observed in the MS/MS spectra [22]. For example, protonated molecule *m/z* 295 [M+H]^+^ ion of peak 100 was observed in MS spectra, and *m/z* 277 [M+H−H_2_O]^+^ ion was exhibited in MS/MS spectra, which corresponded to two fragment ions at *m/z* 280[M+H−CH_3_]^+^ and *m/z* 262 [M+H−H_2_O−CH_3_]^+^, indicating the loss of H_2_O and CH_3_ in the fragmentation pattern. Therefore, tanshinone IIA was unambiguously identified by comparison with the reference standard. Similarly, the [M+H]^+^ ions of cryptotanshinone and isotanshinone IIA, eluting at 75.1 and 76.4 min in ESI-MS/MS spectra, were identified respectively.

### 2.4. Identification of Flavonoid Glycosides in JTT

Flavonoid glycosides are the main constituents originating from *Flos Carthami*. In the full scan mass spectra, most ions were selected to be dissociated with CID in the MS/MS spectra. Although flavonoid glycosides can be ionized under both positive and negative ionization conditions, more relative intense signals were obtained in negative ionization mode. Usually, high abundant quasi-molecular [M−H]^−^ ion was observed in the MS spectra. In the MS/MS spectra, aglycone ions *m/z* 285, *m/z* 301 and *m/z* 287 were finally formed by losing several glycosidic units, which were identified as the characteristic ions of kaempferol, quercetin/hydroxy kaempferol and chalcone, respectively [23,24]. In the present study, a total of 21 flavonoid glycosides were identified or tentatively characterized by comparing their retention times, UV and MS spectra with those of reference standards or through the matching of empirical information with those of published components in the in-house library.

### 2.5. Identification of Cyanogenic Glycosides in JTT

**Figure 4:**
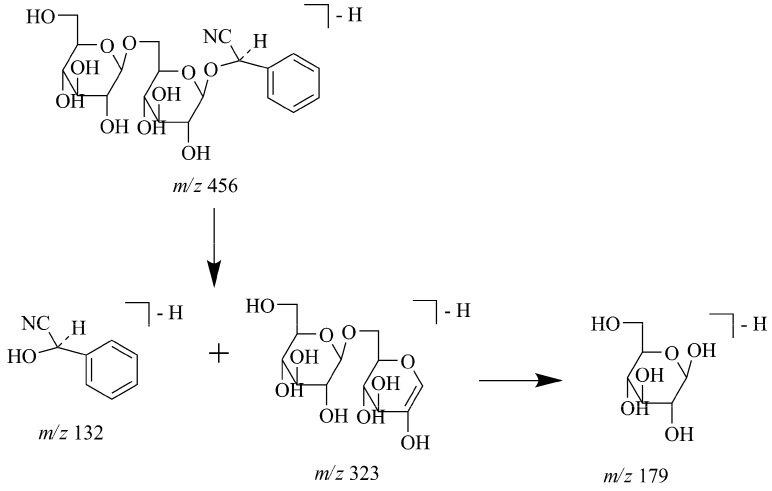
Proposed fragmentation pathway for amygdalin in negative mode.

The UV spectra of peaks 10 and 16 showed maximum absorption bands at 210–230 nm, consistent with the characteristic absorption of cyanogenic glycosides reported in *Semen Persicae* [25]. In the negative ion mode, high abundances of both quasi-molecular ions [M−H]^−^ and [M−H+HCOOH]^−^ were firstly exhibited by these two compounds. In the MS/MS spectra, [M−H−C_8_H_7_NO]^−^ ions were finally formed by loss of neutral fragments mandelonitrile, which was also the characteristic ion of amygdalin and prunasin. In addition, a [M−H−Glc−C_8_H_5_N]^−^ fragment was also observed in MS/MS spectra, according to the proposed fragmentation pathway shown in Figure 4, peaks 10 and 16 were tentatively identified as amygdalin and prunasin, respectively.

### 2.6. Identification of Ginsenosides in JTT

Due to the lack of a conjugated system, no ginsenoside peaks have been found in the UV chromatograms. Therefore, the constituents were characterized mainly based on their mass fragmentation behavior. In the negative ion mode, high abundance quasi-molecular ions [M−H]^−^ and [M−H+HCOOH]^−^ were formed first. In the MS/MS spectra, aglycone ions at *m/z* 475 and 459 were finally formed by losing several glycosidic units, which were also the characteristic ions of panaxatriols and panaxadiols, respectively. Usually, the ginsenosides could be quickly identified by their big molecular weight (>600 Da). However, the comparison with reference standards should also be needed due to the similarity of the structures of some ginsenosides [26]. For example, both peaks 71 and 72 showed quasi-molecular ions at *m/z* 1078 [M−H]^−^ in MS spectra, *m/z* 945 [M−H−(Araf/Xyl)]^−^, *m/z* 783[M−H−(Araf/Xyl)−Glc]^−^, *m/z* 621 [M−H−(Araf/Xyl)−2Glc]^−^ and *m/z* 459 [Agl]^−^ ions could be detected in their MS/MS spectra, which exhibited a fragmentation pathway corresponding to the loss of glycosidic units. The fragment ion at *m/z* 459 corresponding to the protopanaxadiol aglycon moiety was a characteristic ion to identify the structures of these two compounds [27]. By comparison with the reference standards, peaks 71 and 72 were unambiguously identified as ginsenoside Rb2 and Rb3. In addition, Peak 76 showed quasi-molecular ion at *m/z* 955[M−H]^−^ in MS spectra, and exhibited *m/z* 793 [M−H−Glc]^−^, *m/z* 613 [M−H−2Glc−O]^−^ and *m/z* 455 [Agl]^−^ ions in the MS/MS spectra. The ion at *m/z* 455 was produced by successive losing of all linked glucosidic bonds, which was a characterized fragmentation of oleanolic acid type ginsenoside. By comparison with the literature data, the compound was tentatively identified as ginsenoside Ro [28]. With the same method, a total of 15 ginsenosides were tentatively identified with the structurally-relevant product ions.

### 2.7. Identification of 2-(2-Phenylethyl) Chromones in JTT

The UV spectra of peaks 18, 22, 25, 80, 84 and 86 showed two absorption maximum at about 230 nm and 280 nm, consistent with the characteristic absorption of 2-(2-phenylethyl) chromones [29]. 

**Figure 5:**
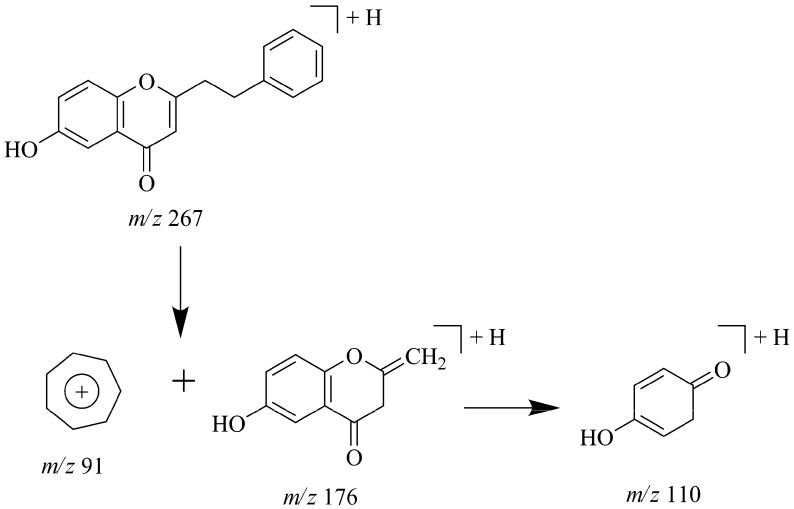
Proposed fragmentation pathway for 6-hydroxy-2-[(2-phenyl)ethyl] chromone in positive mode.

In the full scan mass spectra, [M−H]^−^ of peaks 80, 84 and 86 were observed in negative ion mode, and [M+H]^+^ and [M+H−H_2_O]^+^ in positive ion mode. In the MS/MS spectra, high abundant product ions at *m/z* [M+H−C_7_H_7_]^+^ were formed by the loss of tropylium ions resulted from the RDA cleavage of C ring, while *m/z* [M+H−C_7_H_7_−C_4_H_2_O]^+^ ions were produced by the cleavage of B ring. It could be concluded that C_7_H_7_^+^ and C_4_H_2_O units were the characteristic losses of 2-(2-phenylethyl) chromones in both negative and positive ion mode. By comparison with literature data [30], peaks 80, 84 and 86 were tentatively identified as 6-hydroxy-7-methoxy-2-[(2-phenyl)-ethyl]chromone, 6-hydroxy-2-[(2-phenyl)ethyl]chromone and 6,7-dimethoxy-2-[(2-phenyl)ethyl]chromone, respectively. Additionally, the fragmentation pathway proposed for 6-hydroxy-2-[(2-phenyl)-ethyl] chromone is shown in Figure 5 for the first time.

### 2.8. Identification of Phthalides in JTT

It is reported that two main fragmentation patterns exist in the fragmentation pathway of phthalides: side chain fragmentation reaction corresponding to loss of alkenyl group or ring cleavage corresponding to the loss of H_2_O and carbonyl group [31]. The most abundant product ion at *m/z* 105 was a characteristic ion to identify the structures of these constituents. For example, peaks 42, 49, 79, 83, 87, 96, 98 and 99, which showed fragment ions corresponding to the loss of H_2_O and carbonyl group and gave the characteristic ion at *m/z* 105, exhibited the same fragmentation pathway as phthalides. Among of which, peaks 42 and 49 showed high abundant ion at *m/z* 207[M+H−H_2_O]^+^ in MS spectra, and exhibited fragmental ions at *m/z* 189[M+H−2H_2_O]^+^, *m/*z 179[M+H−H_2_O−CO]^+^, *m/z* 165[M+H−H_2_O−CO−CH_2_]^+^ and *m/z* 145[M+H−2H_2_O−CO_2_]^+^ in MS/MS spectra. According to the law that the *trans*-production peaks eluted early than *cis* peaks in reversed-phase column and comparing with the literature data [32], they were identified as senkyunolide I and senkyunolide H, respectively. By using structurally-relevant product ions [33], peaks 79, 83, 96, 98 and 99 were identified as senkyunolide A, *E*-butenylphthalide, tokinolide B, riligustilide, and levistolide A, respectively. 

### 2.9. Identification of Gingerol-Related Compounds in JTT

The gingerols showed a characteristic UV absorption maximum at 280 and 425 nm, suggesting the presence of an extended conjugation system. It was also found to be useful in the structure confirmation and especially for compounds belonging to homologous series.

Peaks 65, 85 and 95 showed not only quasi-molecular ions at *m/z* [M−H]^−^ in negative mode, but also *m/z* [M+H−H_2_O]^+^ in positive mode, and the fragment ions of both were differentiated by units of 28 Da (C_2_H_4_), which suggested that they were homologues with different alkyl chain length [34]. For example, Peak 65 showed *m/z* 293 [M−H]^−^ and high abundant ionat *m/z* 277 [M+H−H_2_O]^+^ in MS spectra. In the MS/MS spectra, *m/z* 177 [M+H−H_2_O−C_6_H_12_O]^+^ and *m/z* 145 [M+H−H_2_O−C_6_H_12_O−CH_4_O]^+^ were exhibited, which indicated a further fragmentation by the loss of a neutral alkyl moiety and a rearrangement. By comparison with literature data [35], it was tentatively identified as 6-gingerol. Therefore, peaks 85 and 95 were identified as 8-gingerol and 10-gingerol, respectively, by the same method.

For shogaols, high abundant product ions at *m/z* 137[M+H−C_13_H_22_O]^+^ of peaks 89 and 101 were formed in the MS/MS spectra, which indicated the characteristic fragmentation of the keto group on the alkyl chain. By comparison with the literature data [36], peaks 89 and 101 were tentatively identified as 6-shogaol and 10-shogaol, respectively.

## 3 Experimental

### 3.1. Chemicals and Reagents

Reference standards such as danshensu, scopolamine, caffeic acid, amygdalin, hydroxysafflor yellow A, ferulic acid, tetrahydropulmatine, salvianolic acid B, pseudoginsenoside-F11, protopine, costunolide, dihydrocostunolide, cryptotanshinone, tanshinone IIA, ginsenoside Rg1, Re, Rb1, Rb2, Rb3, F1, Rd and Rg3 were purchased from the National Institute for the Control of Pharmaceutical and Biological Products (Beijing, China). 6-Hydroxy-2-[(2-phenyl)ethyl] chromone was isolated and purified from *Lignum Aquilariae Resinatum*, the structure was identified by spectroscopic methods (UV, IR, MS, ^1^H-NMR and ^13^C-NMR), and the purity was determined to be over 98% by HPLC. Acetonitrile and formic acid were of HPLC grade (Merck, Darmstadt, Germany). Ultrapure water from a Milli-Q50 SP Reagent Water System (Millipore Corporation, MA, USA) was used for the preparation of samples and mobile phase. Other reagents were of analytical grade. JTT samples (batch number: 050602) and 15 comprised drugs were kindly offered by National Engineering Research Center for TCM (Shanghai, China).

### 3.2. Sample Preparation

#### 3.2.1. Preparation of Analytical Sample of JTT

JTT and each comprised drug were ground into a fine powder and accurately weighed (400 mg) in a vial. 50% Methanol solvent (4.0 mL) was added and ultrasonic extracted for 30 min to prepare a uniform suspension, then centrifuged at 13,680 g for 10 min (Universal 320R, Hettich, Germany). At last, the supernatant was filtered using a syringe filter (0.22 µm).

#### 3.2.2. Preparation of Standard Solutions

All reference standards were accurately weighed, and dissolved in methanol to obtain stock solutions with proper concentrations (50–1,000 ng/mL). All the stock solutions were stored in the refrigerator at 4 °C until analysis.

### 3.3. HPLC-DAD/ESI-MS/MS

HPLC/DAD analysis was carried out on an Agilent 1100 series HPLC system (Agilent Series 1100, Palo Alto, CA, USA) equipped with a quaternary pump with on-line degasser, auto-sampler, column oven and diode array detector (DAD), scanning from 200–400 nm and the wavelength was then selected and fixed at 280 nm since many peaks could only be found under this condition. Chromatographic separation was performed on a XTerra MS C18 column (5 µm, 4.6 × 250 mm, Waters, Milford, MA, USA) equipped with an XTerra MS C18 guard column (5 µm, 3.9 × 20 mm) (Waters), with the column temperature was set at 25 °C. The mobile phase consisted of 0.1% aqueous formic acid (A) and (B) acetonitrile using a gradient elution of 5–30% B at 0–50 min, 30–100% B at 50–90 min, 100% B at 90–100 min. The flow rate was kept at 1.0 mL/min and 10 µL of sample solution was injected in each run. By solvent splitting, 0.2 mL/min portions of the column effluent were delivered into the ion source of an Agilent LC-MSD Trap XCT mass spectrometer. The MS conditions were fixed as follows: drying gas (nitrogen) flow rate 10 L/min, gas temperature 350 °C, pressure of nebulizer gas 30 psi, HV voltage 3.5 kV, compound stability 100%, threshold 50,000 (ESI^+^) and 10,000 (ESI^−^) and scan range 100–1,500 amu. The amplitude voltage used for fragmentation was 1.0 V. The capillary exit voltage was set at 121 V for both positive and negative ion modes. Positive and negative mode data were acquired using Agilent chemstation software (Agilent Technologies, Palo Alto, MA, USA).

### 3.4. Data Analysis

Data analysis was performed on Microsoft Excel 2003 (Microsoft Corporation). An in-house library was established by searching from online websites such as Google Scholar, PubMed of the US National Library Medicine and Chinese National Knowledge Infrastructure (CNKI) of Tsinghua University, all components of comprised drugs were summarized in a Microsoft Office Excel sheet, which included the name, molecular weight, deprotonated molecular ions, protonated molecular ions, fragment ions and literatures of each published known compound. The “Find” function of Microsoft Office Excel was applied to match the detected mass value with that of published known compounds in the library. The characteristic fragmentation pattern of all components was tentatively elucidated using structurally-relevant product ions.

## 4. Conclusions

In the present study, a reliable and combinative analytical method using HPLC-DAD/ESI-MS/MS was established for rapid identification of multiple constituents in JTT. As a result, a total of 101 constituents were successfully separated and identified, and the fragmentation patterns of nine types of natural products in the complex system were successfully elucidated by the proposed method. 

The present study, compared with the previous studies, showed differences or improvements as follows. First of all, it is the first time a combinative LC/MS (MS^n^) method for screening the chemical constituents in such complex formula comprised of fifteen drugs has been developed. Furthermore, according to the interpretation of their mass behavior obtained from HPLC-DAD/ESI-MS/MS spectra and also taking into account the data provided by the 23 reference standards and the established in-house library, a total of 101 constituents were systematically characterized and identified in a single run. Neutral losses of C_9_H_10_O_3_ and C_9_H_8_O_2_ were the characteristic product ions of scopola alkaloids, and neutral fragment mandelonitrile was the characteristic ion of cyanogenic glycosides. To our knowledge, losses of tropylium ions and C_4_H_2_O unit resulted from the RDA cleavage of C ring existed in fragmentation of 2-(2-phenylethyl) chromones. The results indicated that the developed analysis method could be employed as a rapid, effective technique for structural characterization of chemical constituents in TCMs. This work is expected to provide comprehensive information for the quality evaluation and pharmacokinetic studies of JTT.
